# The Protoxyde of Azote

**Published:** 1868-11

**Authors:** 


THE PROTOXYDE OF AZOTE is being employed as an anæsthetic by Dr. Seymour in the extraction of teeth, producing complete insensibility in two minutes. It is said to be perfectly inocuous, and to be respired without difficulty or repulsion.—*La France Medicale.—Boston Med. Journal.*

---

The oldest Doctor in the world, Professor F. Verdugo, Salamanca, Spain, died, lately, aged 105 years. He had practiced medicine for eighty years.

